# External Use of the Acid Nitrate of Mercury

**Published:** 1855-09

**Authors:** 


### EXTERNAL USE OF THE ACID NITRATE OF MERCURY.

A solution of the nitrate of mercury in strong nitric acid is in very common use at the Hospital for Cutaneous Diseases, and constitutes a very convenient form of caustic. Its formula is—R. Hydrargyri one ounce acidi nitrici (specific gravity 1.50) two ounces; solve. The solution produced is a clear, colorless fluid. The following may be mentioned as some of its chief uses:

*In Carbuncle.*—Mr. Startin usually applies the caustic if the carbuncle be of not more than moderate size, to but one central spot, where it is freely painted for an extent of about a shilling in size. Its effect is to produce an eschar, from beneath which the core afterwards escapes.

*In Acne.*—A very minute drop of the acid is placed by means of a finely pointed glass brush on the apex of any indolent tubercles, whether suppurated or otherwise. It has the effect of opening the pustule, if matter have formed, and if not, induces the disappearance of the induration. The application is followed only by a little smarting pain, and if it has been carefully made, leaves no scar.

*In Boils.*—There can, we think, be little doubt as to the superiority of the caustic treatment over that by the knife, even in the case of very large boils. The pain of the incision, the large sore caused, and the unsightly scar which follows, constitute very formidable drawbacks to a practice for which there is no real necessity. At this hospital, where cases of boils are very common, the knife is never resorted to. The general treatment consists in giving aperients and steel conjointly, and the local, in applying to the apex of the furuncle a full sized drop of the acid nitrate solution. The morbid action generally terminates coincidentally with the application, and the core is thrown off through a comparatively small opening, the resulting cicatrix being insignificant.

*In Lupus.*—The acid nitrate is one of the most efficient and convenient forms of caustic in this disease. Mr. Startin does not, however, employ it solely, but uses also the biniodide of mercury, and a paste, of which arsenic is the principal ingredient. The acid nitrate is chiefly used in indolent tubercles, and to indurated patches not actually ulcerated. After ulceration has occurred the arsenical paste is preferred.

*For Sloughing Ulcers.*—The practice of treating unhealthy ulcerations wherever situated, by means of caustics, is much pursued at this hospital, and with excellent results. The pain attending the application of nitric acid has been much overrated by the profession generally, and its use has consequently been avoided in many instances in which it would have been found efficient to completely change the course of the morbid action and induce healthy processes. Its powers in cases of phagedæna are now widely recognized, and its use will probably soon extend to various other kinds of ulceration of somewhat similar nature, but much less severity. The pain spontaneously caused by an unhealthy sore during a single night is probably much more than that produced by an application of caustic. In most cases of unhealthy ulcers, Mr. Startin employs either the solution of the acid nitrate or the arsenical paste just referred to. The rapidity with which the surface granulates afterwards is often surprising.

*In Moles, Nævi, &c.*—Small moles on the face. if superficial and not too thick, may be readily destroyed by the acid nitrate. A cicatrix of course results, but it is small, and far less unsightly than the original disease. Small cutaneous nævi are often treated both at this and the various other London Hospitals, by means of the nitric acid. Unless the disease be of very small extent, the employment of a ligature appears to be a much more certain means of effecting the end desired. If there be a subcutaneous base to the morbid structure it often persits in growing, despite frequent applications of escharotics. There is a mild form of dilated cutaneous capillaries which produces the marks known as "port-wine stains," "spiders," etc., in the treatment of which much benefit may be obtained by the dexterous application of fluid caustics. With a finely pointed glass, charged either with nitric acid or the acid nitrate of mercury, the tortuous vascular trunks should be severally painted, a minute streak of the caustic being thus left along the whole course. In this way, by repeated applications, the whole of the larger vessels may be destroyed, and the disfigurement, to a large extent, diminished. The "port wine stain" is of course very much more difficult to remove than the less diffused forms of this condition, such, for instance, as are of frequent occurrence on the cheeks or nose; even in it, however, much benefit may by patient treatment be gained.—*Medical Times and Gaz.*

